# Marginal effects of economical development and university education on China’s regular exercise population

**DOI:** 10.3389/fpubh.2024.1411191

**Published:** 2024-07-15

**Authors:** Agudamu, Te Bu, Yang Zhang

**Affiliations:** ^1^Graduate School of Social Welfare, Sungkyunkwan University, Seoul, Republic of Korea; ^2^College of Physical Education, Hunan Normal University, Changsha, China; ^3^HEHA CAT Sports Science and Technology Institute, Changsha, China; ^4^Independent Researcher, Windermere, FL, United States

**Keywords:** educational attainment, exercise, GDP, inequality, physical activity, weather

## Abstract

**Objective:**

Although the regular exercise population is a key metric for gaging the success of China’s fitness-for-all activities, effective policy approaches to increase mass sports participation remain unclear. Previous research suggests that GDP, educational attainment, sports resources, and meteorological conditions could influence regular exercise participation. Therefore, this study first analyzed the macro-level correlates influencing China’s regular exercise population.

**Methods:**

We utilize ordinary least squares (OLS) regression and geographical weighted regression (GWR) to theorize the relationship. The analysis encompasses data from the 31 administrative regions of Mainland China, as reported at the end of the 13th Five-Year Plan period. The log–log model enables us to quantify the marginal effect (elasticity) of the explanatory variables.

**Results:**

The OLS regression showed that regional GDP and the proportion of the population with a university education were significant predictors. In the global model, the marginal effects of regional GDP and university education were 0.048 and 0.173, respectively. Furthermore, the GWR revealed a distinct geographic pattern that corresponds to the classic Hu Line.

**Conclusion:**

While regional GDP was also a significant correlate in our model, the elasticity demonstrates that university education had an asymmetric effect on China’s regular exercise population. Therefore, this paper sheds light on a policy priority for the upcoming 15th Five-Year Plan, emphasizing the strategic importance of expanding university education to enhance mass sports participation. In turn, a better-educated populace may yield significant secondary effects on public health and contribute to the high-quality development of the Chinese path to modernization.

## 1 Introduction

The concept of modernization, emerging in the mid-twentieth century, is generally regarded as “a multifaceted process involving changes in all areas of human thought and activity” (1). As the post-World War II era ushered in a period of peace and the industrial civilization progressed, GDP growth became a universal benchmark for modernization. In the context of China, modernization is seen as a blend of contemporary values with the enduring essence of traditional Chinese culture. A notable theoretical advancement in this concept is Xi Jinping’s assertion that “people’s health is the primary indicator of modernization.” This underscores the importance of the proactive health concept (2), with China’s fitness-for-all activities set to play a key role in the Chinese path to modernization (3). Since its inception in 1995, the National Fitness Plan has shown promising results, with recent data revealing that 37.2 percent of China’s population regularly do physical exercise (4). However, this figure falls short when compared to the 40–45 percent participation rates observed in developed countries (5), indicating that China still faces significant challenges in promoting its mass sports program. The latest National Fitness Plan (2021–2025) highlights the government’s focus on improving the quality of fitness-for-all activities. It points out ongoing challenges, such as the imbalanced regional development of these activities and the insufficient provision of public fitness services. Therefore, addressing the issue of imbalanced regional development is essential in the next phase of the nation’s modernization endeavors.

The developmental goals of the National Fitness Plan are articulated through several critical metrics designed to gage its success. These metrics encompass the percentage of the population that regularly do physical exercise (thereafter, regular exercise population), the establishment of 15-min community fitness circles, the number of social sports instructors, and, on a broader scale, the passing rate of the national physical fitness standard. Thus, employing quantitative research focused on these four key metrics can provide policy recommendations for drafting a refined National Fitness Plan. The existing literature has largely focused on regional disparities in passing rates, with limited discussion given to the regular exercise population (6)—arguably the most important metric for assessing the effectiveness of fitness-for-all activities. To date, there has been no quantitative analysis examining the imbalanced regional development concerning the regular exercise population.

Previous research on correlates and determinants influencing physical exercise practices has centered on individual-level factors, such as age, sex, and income (7). While these studies are valuable for influencing individual behavior, they provide limited guidance for developing effective national policies that target the entire population, especially for a diverse and populous nation like China. From a macro perspective, existing theories focus on four key areas: economic fundamentals, health consciousness, resource availability, and climatic factors. First, economic development plays a critical role in facilitating the mass sports program. The level of economic prosperity determines the extent of government support for non-essential social activities, such as fitness-for-all activities. Empirical data from a study conducted in 34 nations revealed a positive correlation between *per capita* GDP and economic freedom and increased engagement in physical exercise (8). In the meantime, an individual’s participation in leisure-time physical exercise is influenced by their economic status. A pooled analysis of health surveys conducted in England in 2008, 2012, and 2016 revealed that individuals from high-income households are more likely to participate in and spend more time engaging in moderate-to-vigorous physical exercise compared to those from lower-income households (9). Similarly, data from Belgium indicated that sports consumption and participation were stronger among higher-income households (10). Essentially, the inequality in sports participation can be attributed to a better economic position, which stimulates proactive health awareness (11).

Second, education not only critically influences employment opportunities but also affects participation in physical exercise at the population level. For example, an analysis of physical exercise patterns across 27 European countries revealed a strong correlation between higher government expenditure on education, as a proportion of GDP, and a greater likelihood of citizens engaging in regular exercise (12). The said study additionally found that government health spending does not directly impact exercise habits, suggesting that health promotion policies may be more effective when focused on education. Similar studies conducted in Brazil, China, Ireland, Sweden, and the United States reinforce education as an important correlate of sports participation (13–17). The mechanism is suggested to be that better education affects an individual’s economic status and health beliefs, which in turn shape their long-term attitude toward physical exercise.

Third, government infrastructure spending on sports venues has an important role in promoting public exercise behavior. In this regard, Dallmeyer and colleagues showed that in Germany, the key factor is not the average fiscal expenditure but rather consistent fiscal support (18). To effectively promote public health policy through sports, it is imperative for governments to consistently provide funding for the development of sports resources to influence citizens’ exercise habits. Similarly, data from 21 European countries endorse the notion that an accountable government (e.g., through investing in sports venues) positively impacts the physical exercise levels of its citizens (19). In developing countries, the availability of sports resources has an even greater impact on public interest and participation in sports. In a populous country like China, the *per capita* availability of sports resources is a limiting factor in organizing mass sports events. Of note, it is important to consider availability in a broader sense, as research from both developing and developed countries indicates that individuals are less inclined to use sports venues that are located far from their homes (20, 21).

Fourth, natural factors can restrict sporting activities to some extent. Obradovich and Fowler reported that recreational physical activity decreases during both cold and extremely hot conditions, as well as on days with precipitation, based on data collected from over 1.9 million survey respondents in the United States (22). Given that global warming appears to be beyond mitigation under current global efforts (23), it is likely that heat will have a greater impact on global sports participation going forward. Additionally, meteorological conditions caused by human activities, such as air pollution, would also have a detrimental effect on sports participation (24). Given China’s vast territory, which spans several climatic zones, the impact of meteorological conditions on physical exercise is an aspect that cannot be overlooked.

Based on actual statistics regarding China’s regular exercise population (as presented in the following section), it becomes evident that the aforementioned macro factors are intricately interconnected. For instance, Guangdong consistently ranks highest among provinces in terms of GDP output. It boasts remarkable sports resources, particularly in professional soccer and basketball, and benefits from a favorable climate. However, its regular exercise population is among the lowest. In contrast, Liaoning faces freezing temperatures in winter, and its economic development is constrained by its geographical location. Despite these challenges, Liaoning enjoys one of the largest regular exercise populations. Such a phenomenon, in some cases against popular theories (22), not only underscores regional disparities in the regular exercise population but also highlights a discrepancy between the real conditions and the theoretical framework. This disparity underscores the imperative of quantifying the macro factors contributing to these variations.

From a macro perspective, the birth rate in China is concerning, and the Chinese society as a whole is experiencing accelerated aging. By 2050, it is expected that the percentage of individuals aged 55 to 64 in the overall workforce will increase to 26.7% (25). This unfavorable demographic structure underscores the urgency to promote the proactive health concept, particularly through regular exercise participation, to enhance health outcomes and alleviate both personal and national financial burdens associated with healthcare costs. A recent study reinforces this notion. Analysis of credit utilization data from one million Chinese between 2018 and 2021 revealed a significant inverse correlation between individual sports consumption and medical expenses: for every 1 % increase in sports consumption, there was a corresponding decrease of 0.203 percent in personal medical expenditure (26). The benefits of regular exercise participation on health and financial resilience cannot be overstated.

Therefore, the purpose of this paper was to identify macro correlates that can explain the dynamics of China’s regular exercise population. Here, we hypothesize that China’s regular exercise population is influenced by four macro factors: economic, educational, resource-related, and meteorological. Their relationship can be mathematically represented in Eq. (1) as follows:


(1) $$\begin{array}{l} \ln\left(\begin{array}{l} \mathrm{regular}\;\\ \mathrm{exercise}\;\\ \mathrm{population} \end{array}\right)=\ln\left(\mathrm{economy}\right)+\ln\left(\mathrm{education}\right)+\\ \ln\left(\begin{array}{l} \mathrm{sports}\;\\ \mathrm{resources} \end{array}\right)+\ln\left(\mathrm{climate}\right)+\varepsilon \end{array}$$

where ε follows a parametric probability distribution.

Moreover, this paper utilized modern econometric and spatial statistical methods to deepen the understanding of how the elasticity of these macro factors varies across different regions. To compute the marginal products of these macro factors, we apply the natural logarithm function to the data, as illustrated in Eq. (1). These marginal products are referred to as marginal effects within the context of our paper to better match the specific focus of our research. Essentially, we advocate for a macro-level theory of human behavior that is consistent with realities. The insights gained from this analysis are intended to provide policymakers with a solid foundation for crafting upcoming national policies.

## 2 Methods

### 2.1 Data overview

According to the General Administration of Sport of China, “population regularly do physical exercise” is defined as engaging in physical exercise at least three times a week, with each session lasting 30 min or more, and at a moderate or higher intensity level. The study examined the regular exercise population across 31 administrative regions in Mainland China, using government statistics collected between 2020 and 2021 as the response variable, except for Tibet, which only had data available for 2019. These statistics reflect the overall progress of the fitness-for-all activities by the end of the 13th Five-Year Plan (2016–2020).

The explanatory variables encompass four macro factors discussed previously. First, regional GDP can serve as a composite indicator to approximate the level of fiscal expenditure for sporting causes, as well as its impact on human development (27). It is worth noting that the author (Y.Z.) traditionally does not consider *per capita* GDP for scientific analysis. Although there may be some controversy surrounding the 2015 income Gini coefficient of 0.62 obtained from the China Household Finance Survey (28), its primary conclusion remains indisputable: wealth inequality in China has always existed and is significantly greater than that observed in OECD countries. According to the Global Wealth Report 2023 (29), the wealth Gini coefficient in Mainland China has increased to 0.707, and the share of the top 1% of Chinese residents in total wealth has risen to 31.1%, indicating a further widening of wealth inequality. Given that Chinese individuals possess the highest savings rate globally, it is reasonable to question the potential distortion in the *per capita* GDP data. Furthermore, better education is often associated with improved job prospects, making it more appropriate to utilize education as a proxy measure of an individual-level economic standing (30).

Second, despite the popularity of certain leisure physical activities in China, such as fitness walking and square dancing, which do not necessarily depend on traditional sports venues, the *per capita* sports area remains an important metric for assessing the resources available for fitness and sports activities. Moreover, the accessibility to sports venues significantly affects an individual’s likelihood of participating in sports (31). Hence, both the *per capita* sports area and the number of public buses per 10,000 people were analyzed as correlated indicators of sports resources for physical exercise.

Third, statistics on educational attainment from the 2020 Seventh National Population Census were examined to understand the impact of education on sports participation (32). To explore the sensitivity of various levels of educational attainment, the data were decomposed into five categories: population with a university education, population with a tertiary education, population with at least high school education, population with at least middle school education, and population with at least primary school education.

Fourth, meteorological data from 2016 to 2020 were considered to explore factors relevant to exercise behaviors, including the average daily temperature, the average highest daily temperature, annual precipitation (measured from 0800 to 2000 h), the number of rainy days, haze days, dust and sand storm days per year, and the number of days with extreme highest temperatures (≤ 5°C and ≥ 35°C). Meteorological data sets were collected by over 100 benchmark weather stations in China.

Table 1 presents the primary data used in the statistical modeling, offering insights into the macro factors influencing China’s regular exercise population. The full data source is accessible on Figshare.^1^

**Table 1:** Descriptive data of main interests.

Administrative region	Regular exercise population (%)	Regional GDP (100 million CNY)	Population with a university education (%)
Beijing	50.18	36102.55	28.59
Tianjin	45	14083.73	15.96
Hebei	43.41	36206.89	5.35
Shanxi	37.07	17651.93	8.01
Inner Mongolia	35.54	17359.82	8.71
Liaoning	46.49	25114.96	9.37
Jilin	37.5	12311.32	9.21
Heilongjiang	36	13698.5	7.47
Shanghai	45.7	38700.58	21.52
Jiangsu	40.3	102718.98	9
Zhejiang	42.2	64613.34	8.51
Anhui	36.2	38680.63	6.05
Fujian	41.3	43903.89	7.16
Jiangxi	37.6	25691.5	5.17
Shandong	41	73,129	6.65
Henan	36.5	54997.07	4.9
Hubei	33.07	43443.46	7.37
Hunan	38.51	41781.49	5.14
Guangdong	35.65	110760.94	7.11
Guangxi	39.8	22156.69	4.75
Hainan	33.34	5532.39	6.6
Chongqing	47.65	25002.79	7.25
Sichuan	33.7	48598.76	6.04
Guizhou	35.5	17826.56	5.37
Yunnan	36.01	24521.9	5.51
Tibet	29.6	1902.74	5.84
Shaanxi	43	26181.86	8.77
Gansu	36.9	9016.7	6.79
Qinghai	35.8	3005.92	7.07
Ningxia	36.2	3920.55	8.38
Xinjiang	30.89	13797.58	6.74

### 2.2 Spatial statistics

**Figure 1:**
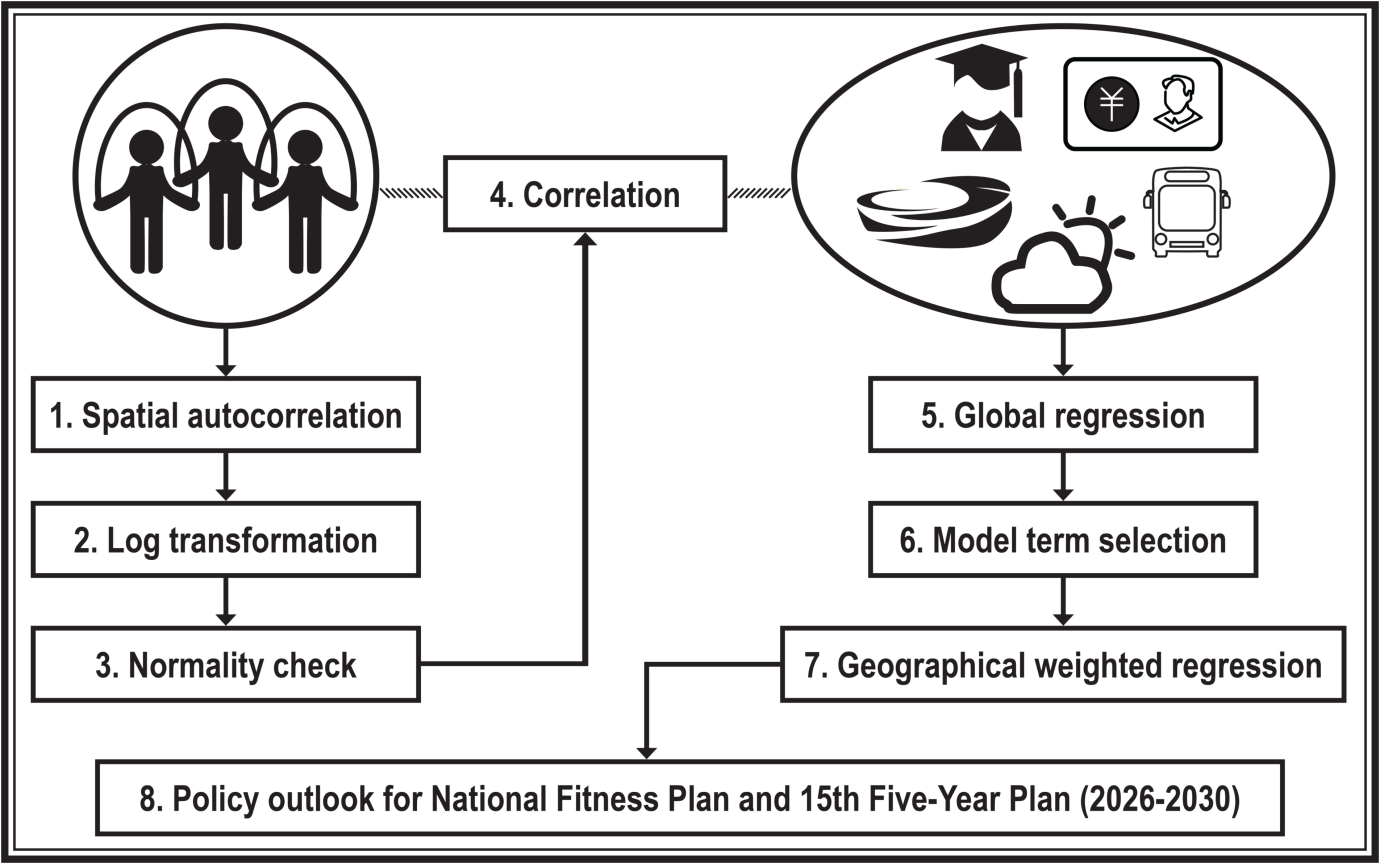
Flowchart of the study.

The statistical modeling was conducted using ArcGIS Pro version 3.2.1 (Esri Inc., Redlands, CA, United States) and the gamlss package version 5.4–20 in R. For brevity, we have omitted the presentation of commonly calculated equations in computer software and domain-specific statistical theories. Figure 1 illustrates an overview of the steps involved in the statistical modeling.

During the estimation period, we performed preliminary statistical analyses. Our first step involved utilizing the global Moran’s I statistic to evaluate spatial autocorrelation of regular sports populations across the 31 administrative regions. Following this, we converted the raw data into natural logarithms. In instances where the data frame contained zero or negative values, we applied a log-modulus transformation, which shares similar characteristics with a traditional log transformation. Then, we assessed whether the log-transformed response variable adhered to a normal distribution. We also carried out a Pearson correlation analysis to identify significant correlations between the response variable and potential explanatory variables. Only those variables that demonstrated significant correlations were selected for inclusion in the subsequent regression analysis.

In the search for sensible model terms for the ordinary least squares (OLS) regression, we adopted a forward selection approach, guided by statistical significance and the Akaike Information Criterion (AIC). Briefly, one explanatory variable was incrementally included in the OLS model until all terms were shown to be significant predictors (Chi-square *p* < 0.05) of the response variable, and no more improvement in AIC was possible. Of note, the statistical significance was determined by the generalized likelihood ratio test statistic, which, in general, is preferable to the Wald test statistic. Additionally, we experimented with fitting model terms using the Generalized Additive Model for Location, Scale, and Shape (GAMLSS). This method involved the use of a non-parametric smoother within a normal distribution family, aiming for a more flexible model adaptation to the data.

The model terms identified as significant in the global model were subsequently incorporated into a geographical weighted regression (GWR), applying a Bisquare weighting scheme to account for geographical variations. Finally, the adequacy of the GWR model specification was assessed by analyzing the residual spatial autocorrelation, ensuring that our model accurately reflected the underlying spatial patterns in the data.

## 3 Results

### 3.1 Preliminary analyses

The global Moran’s I statistic, with a z-score of 3.51 (*p* < 0.001), rejects the hypothesis of complete spatial randomness in regular sports populations across the 31 administrative regions. Furthermore, the Shapiro–Wilk normality test for the log-transformed regular sports populations yielded a *p*-value of 0.565. These preliminary statistics justify both the global and local linear modeling.

Pearson correlation analysis revealed moderate yet significant correlations between regular sports populations and several explanatory variables: regional GDP (*r* = 0.40, *p* < 0.05), educational attainment (*r* = 0.53–0.62, with all *p*-values below 0.01), and the number of public buses per 10,000 people (*r* = 0.6, *p* < 0.001). Accordingly, *per capita* sports area and all meteorological variables were deemed statistically insignificant for inclusion in further modeling.

### 3.2 OLS model

Table 2 presents the stepwise term selection procedure. We began with a model containing only regional GDP. Given its statistical significance, this term was considered for retention in the final model. Subsequently, we incorporated each of the incremental models representing different levels of educational attainment. Upon including the population with a university education (Chi-square *p* < 0.001), the AIC of model 2 decreased to −52.8, indicating a substantial improvement in model performance. This process was repeated for subsequent models. However, as we moved toward lower levels of educational attainment, the AIC values for models 3–6 gradually increased, and the associated terms became statistically insignificant. Consequently, the population with a university education was retained at this stage.

**Table 2:** Model significance, performance, and fit from OLS and GAMLSS models.

Model no. and specification	Generalized likelihood ratio test statistic					F-statistic	AIC	R^2^
	ln(econ.)	ln(edu.)	ln(bus)	CSln(econ.)	CSln(edu.)			
M1 = ln(econ.)	5.49^*^	–	–	–	–	5.62	−40.8	0.162
M2 = ln(econ.) + ln(edu.^1^)	7.11^**^	14.03^***^	–	–	–	12.28	−52.8	0.467
M3 = ln(econ.) + ln(edu.^2^)	5.77^*^	12.65^***^	–	–	–	11.14	−51.5	0.443
M4 = ln(econ.) + ln(edu.^3^)	2.57	11.83^***^	–	–	–	10.48	−50.6	0.428
M5 = ln(econ.) + ln(edu.^4^)	0.89	8.34^**^	–	–	–	7.87	−47.1	0.360
M6 = ln(econ.) + ln(edu.^5^)	0.81	5.62	–	–	–	6.04	−44.4	0.301
M7 = ln(econ.) + ln(edu.^1^) + ln(bus)	5.11^*^	2.91^#^	0.28	–	–	8.05	−51.1	0.472
M8 = CSln(econ.) + CSln(edu.^1^)	–	–	–	13.98^**^	20.33^***^	–	−53.0	0.640

AIC, Akaike information criterion; bus, public buses per 10,000 people; CS, cubic splines; eco., regional GDP; edu., educational attainment. ^#^, *, **, and *** denote Chi-square p-values equal to or less than 0.1, 0.05, 0.01, and 0.001, respectively. edu^1^, population with a university education; edu^2^, population with a tertiary education; edu^3^, population with at least high school education; edu^4^, population with at least middle school education; edu^5^, population with at least primary school education.

Further, we introduced the number of public buses in model 7. Although the inclusion of this term slightly increased the AIC compared to model 2, the added term did not significantly contribute. While the R^2^-value suggested an improved model fit, this could potentially be attributed to a coincidental correlation with the response variable, thus, we opted for model parsimony and dropped the number of public buses from the model terms.

Additionally, we re-fitted model 2 with a non-parametric smoother in the GAMLSS model 8. Through experimentation, penalized cubic splines marginally decreased the AIC and substantially enhanced the model fit compared to the parametric linear model 2. It is necessary to remind that this model is entirely experimental and is further discussed in a later section.

Finally, we examined the residuals of model 2. The Jarque-Bera statistic yielded a *p*-value of 0.896, suggesting the absence of model bias and confirming that the model is properly specified. Hence, based on considerations of statistical significance, AIC, and R^2^-value, model 2 was determined to be the most suitable assessment of China’s regular sports population. The global model can be mathematically expressed in Eq. (2) as follows:

(2) $$\begin{array}{l} \ln\left(\begin{array}{l} \mathrm{regular}\;\\ \mathrm{exercise}\;\\ \mathrm{population} \end{array}\right)=2.811\;\left[0.143\right]+0.048\;\left[0.014\right]\;*\ln\left(\begin{array}{l} \mathrm{regional}\;\\ GDP \end{array}\right)+\\ 0.173\;\left[0.024\right]*\;\ln\left(\begin{array}{l} \mathrm{population with}\;\\ a\;\mathrm{university education} \end{array}\right) \end{array}$$

where, robust standard errors in [].

### 3.3 GWR model

With a distance band of 3,553,403 meters, the AIC (small sample adjusted) of the GWR model was −50.11. While this value increased slightly compared to the OLS model 2, their difference was less than 3. Meanwhile, the model fit improved to 0.508 from 0.467 of model 2, highlighting the advantages of transitioning from a global model to a local model. Additionally, the z-score of the regression residuals from the spatial autocorrelation was −0.135 (*p* = 0.893), indicating that the GWR model was spatially random and correctly specified.

The GWR model generated local models for each administrative region, as summarized in Table 3. While the local marginal effects fluctuate across regions, the main point remains evident: university education had a much more pronounced marginal effect than regional GDP (mean ratio of university education to regional GDP = 4.43). It is worth noting that the local model fit decreased in Fujian, Guangdong, Guangxi, and Hainan.

**Table 3:** Estimates of coefficients from the GWR model.

Administrative region	*β*econ.	*β*edu.	Local R^2^
Beijing	0.039	0.167	0.466
Tianjin	0.038	0.166	0.461
Hebei	0.039	0.167	0.464
Shanxi	0.040	0.169	0.468
Inner Mongolia	0.043	0.170	0.485
Liaoning	0.037	0.163	0.456
Jilin	0.039	0.163	0.464
Heilongjiang	0.046	0.164	0.487
Shanghai	0.030	0.161	0.424
Jiangsu	0.032	0.162	0.434
Zhejiang	0.030	0.160	0.417
Anhui	0.033	0.163	0.434
Fujian	0.029	0.160	0.406
Jiangxi	0.032	0.162	0.418
Shandong	0.036	0.164	0.449
Henan	0.037	0.166	0.451
Hubei	0.037	0.166	0.442
Hunan	0.035	0.164	0.428
Guangdong	0.031	0.160	0.397
Guangxi	0.036	0.163	0.410
Hainan	0.031	0.155	0.352
Chongqing	0.039	0.168	0.449
Sichuan	0.043	0.172	0.462
Guizhou	0.039	0.166	0.434
Yunnan	0.042	0.168	0.429
Tibet	0.051	0.194	0.461
Shaanxi	0.041	0.170	0.468
Gansu	0.047	0.178	0.496
Qinghai	0.049	0.182	0.494
Ningxia	0.044	0.173	0.482
Xinjiang	0.053	0.216	0.504

β, coefficient; eco., regional GDP; edu., population with a university education.

**Figure 2:**
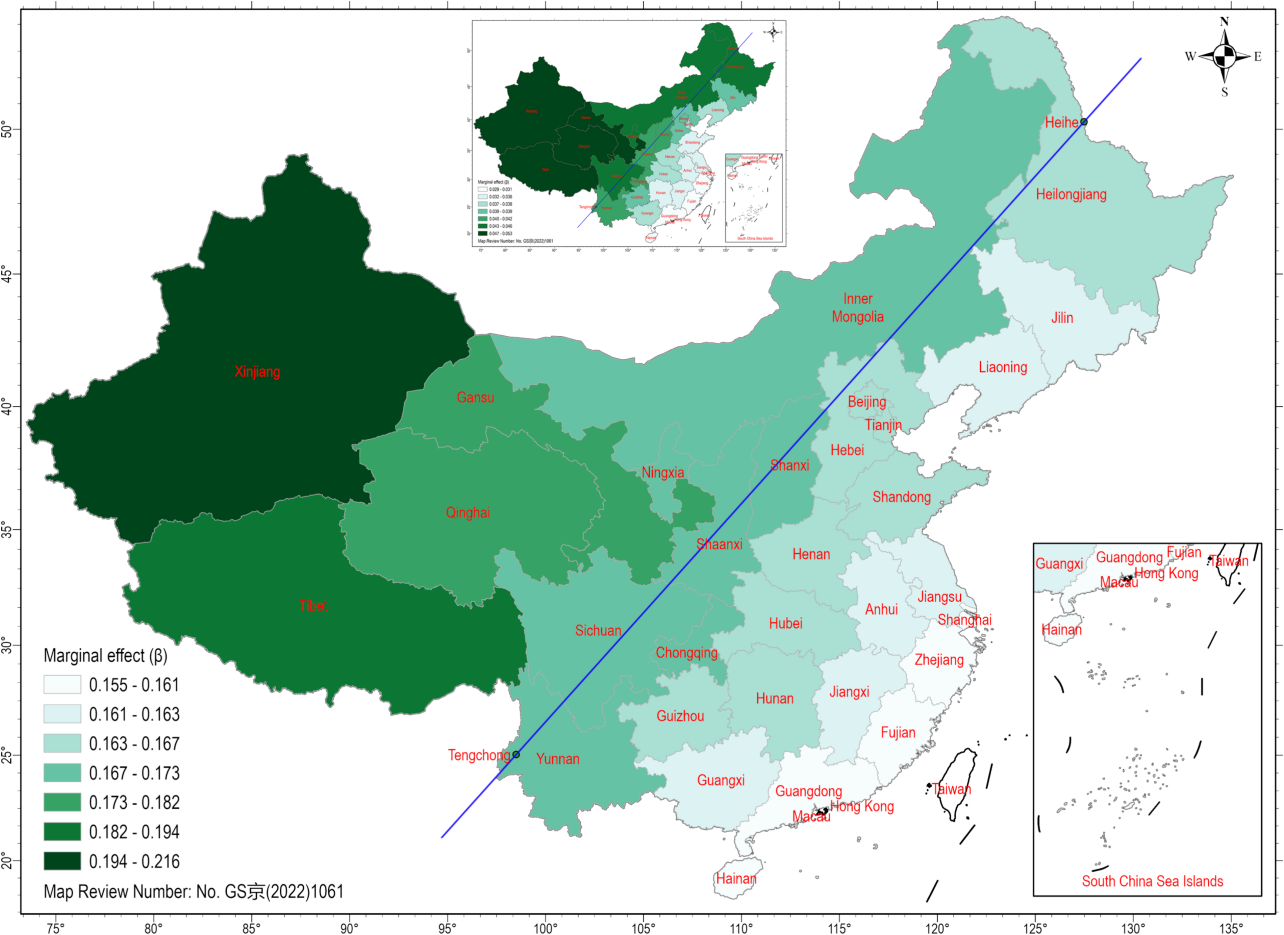
Spatial distribution of GWR model coefficients. The main map highlights the marginal effect of university education on China’s regular sports population, while the scaled map illustrates the marginal effect of regional GDP. The magnitude of the coefficients is shown by the gradient green color: the darker the shade, the greater the coefficient. The Hu Line is represented by the blue line.

We visualize the local marginal effects across the 31 administrative regions in Figure 2. The local marginal effects of educational attainment exhibited a notable disparity between the west and east sides of the Hu Line (33). In other words, a one-unit increase in the proportion of the population with a university education in Western China could lead to a greater increase in the regular exercise population compared to Eastern China. Similarly, the local marginal effects of regional GDP displayed a comparable pattern, as depicted in the scaled map.

## 4 Discussion

The National Fitness Plan, alongside existing theories, suggests that the regular exercise habits of the population may be influenced by factors related to economics, education, resources, and meteorological conditions. In this paper, we estimated Eq. (1) through the use of both global and local regressions. Effectively, the work presented is consistent with a methodology to let the actual data inform the theoretical relationship. Our findings reveal that China’s regular exercise population did not significantly react to the emphasis on sports resources outlined in the National Fitness Plan or to meteorological conditions. As demonstrated in Eq. (2), our log–log model leads to the identification of elasticity for the two significant macro factors, with a particular emphasis on university education. The significance of this paper extends beyond being merely the first quantitative analysis of the key metric defined in the National Fitness Plan. The insights provided offer guidance on more effective resource allocation strategies for the Chinese path to modernization and could influence the formulation of China’s 15th Five-Year Plan.

The first obvious question is whether the observed statistical correlation, especially between educational attainment and participation in sports, reflects a genuine causal relationship. In our view, educational attainment plays a critical role in determining exercise habits. This view is supported by a large body of empirical research. Here, we provide just a handful of examples from studies conducted in China. Hui and colleagues found that diabetic adults with a university education not only had a better understanding of physical activity but also participated in it more frequently (34). Similarly, Li and colleagues conducted a 4-year cohort study to investigate the risk factors for physical inactivity. Their research found that, compared to those with a university education, the population with only a primary school education were 2.36 times more likely to lead a physically inactive lifestyle, while those with a middle/high school education were 2.13 times more likely to be inactive (35). Moreover, an analysis of four national surveys on fitness-for-all activities revealed a decline in regular exercise among individuals with lower levels of education (i.e., primary and middle school). In contrast, the most significant increase in regular exercise was seen among individuals holding a master’s degree or higher (36).

Regarding the impact of the economy on the demographic dynamics of sports participation, established theories provide insights (8). Two principal theories explain the role of education in this context. The first theory suggests that education leads to a shift in health perception, thereby influencing an individual’s lifelong attitude toward well-being (37). We refer to it as the education-health conscientiousness pathway. The second theory emphasizes the link between an individual’s economic status and their education. According to this view, educational attainment directly affects employment prospects, which in turn shapes the individual’s economic base (38). This relationship is termed the education-employment pathway here. Additionally, a third perspective integrates both of the aforementioned theories. We propose that university education not only fosters a heightened awareness of health during one’s academic years but also paves the way for wealth accumulation in the future. This holistic view underscores the multifaceted impact of education on both health consciousness and economic security, suggesting a synergistic effect on sports participation.

Two important aspects need emphasis. Our combination model demonstrates that educational attainment, particularly at the university level, significantly impacts regular exercise participation compared to tertiary education. This implies that obtaining a university degree increases the likelihood of engaging in physical exercise throughout one’s life. Tertiary education encompasses formal university academic programs and vocational programs preparing individuals for the workforce. In China, vocational education lasts 5 years, including 3 years of high school education, while university education spans 4 years. Though vocationally educated individuals may have less exposure to non-vocationally related health education programs due to a two-year education gap, we believe that education’s influence primarily stems from the education-employment pathway mentioned earlier. Evidence suggests concerning education quality among vocational school students, with their career prospects not matching those of university graduates (39). Consequently, changes brought about by vocational education may only meet basic living needs, limiting an individual’s overall future development. Further investigation is necessary to validate our hypothesis.

Furthermore, priority for future intervention should be given to the western part of China, specifically the area west of the Hu Line. The Hu Line, first delineated by Prof. Hu Huanyong in 1935 (33), serves as the demarcation line for China’s population distribution. Despite facing nearly a century of scrutiny, it continues to be supported by a wealth of economic development data. Economic growth east of the Hu Line is significantly more diverse compared to the western region due to differences in environmental conditions and population distribution. Our findings indicate that sports development also aligns with the Hu Line. According to our model, the western part of China lags behind other regions in terms of both economic development and educational attainment. From a policy-making perspective, our findings suggest that relying solely on sports policy will not immediately address the disparity between the east and west. Instead, it is crucial to prioritize national-level economic development, particularly the promotion of science education, to bring about meaningful change.

While our analysis does not demonstrate a significant relationship between sports resources and the regular exercise population, it is still valuable to discuss the underlying causes. We argue that two factors contribute to this occurrence. Firstly, sports resources are primarily allocated to urban areas (40). According to the findings of the Seventh National Population Census, more than one-third of China’s population resides in rural areas. Therefore, the *per capita* sports area does not accurately reflect the quantity of sports resources available to the rural population. Additionally, while the availability of sports venues is essential for promoting fitness-for-all activities, the economic foundation, particularly time resources, also plays a crucial role in influencing individuals’ inclination to use sports venues. The consumption of non-essential sports is influenced by income (41), meaning that having more sports resources may not necessarily lead to increased use of sports venues in areas with significant wealth gaps. Moreover, research indicates that exercise participation declines significantly when sports venues are located more than 10–15 min away from one’s residence (31). Thus, the availability of sports resources does not effectively contribute to the growth of China’s regular exercise population at the current stage. Chinese authorities are aware of this issue, and the current National Fitness Plan prioritizes the establishment of 15-min community fitness circles. The penetration rate of community gyms in Changsha, as demonstrated by our HEHA CAT Fitness model, is five times greater than that of traditional gyms (42). Future research could potentially validate the correlation between the regular exercise population and the number/length of greenway trails since fitness walking is the most common physical exercise in China.

It is also useful to discuss the goodness of fit of the model. This paper aims to present an explanatory model, making the R^2^-value irrelevant for this purpose. Through GAMLSS analysis, we showcase the ability to overcome the limitations of standard OLS fitting using a modern regression approach, which is valuable for predictive models. However, it is worth noting that in Southern China (i.e., Guangdong, Guangxi, and Hainan), the R^2^-value was notably lower than the national average. In Hainan, it was more than two standard deviations below the average. Notwithstanding that additional factors may affect exercise participation in these regions, an inherent limitation of the methodology used in this study may prevent us from reaching an accurate conclusion, at least within a few local extreme contexts. Specifically, even though we believe the observed relationships, such as the effect of educational attainment, may indicate a genuine cause-and-effect relationship, our theoretical model can only establish correlations with exercise behaviors in strict statistical terms. Put simply, neither the local GDP nor university education can be considered determinants of China’s regular exercise population. Similarly, we cannot rule out the existence of a true causal relationship between meteorological conditions and regular exercise participation. Using the aforementioned R^2^-value outliers as an example, we hypothesize that high temperatures may be a potential cause of this occurrence locally. In Chinese culture, individuals often avoid exercising under direct sunlight. Given that these three regions experience the highest number of sunshine hours in China, they are likely affected by intense sunlight. This suggests that the impact of climate on exercise habits may vary (22), and extremely high temperatures influence the level of physical exercise most among the Chinese population. Further empirical research is necessary to delve deeper into our hypothesis.

Moreover, although this study analyzed a large amount of high-quality meteorological data, the regular exercise population data was based on government surveys instead of empirical data. Hence, there could be discrepancies compared to results from controlled experimental studies. Indeed, although this study did not find any influence of haze days or dust and sand storm days on regular exercise participation, other empirical studies suggest that both of these meteorological conditions negatively impact individual-level exercise participation (43). Nevertheless, although research into sports participation has burgeoned in the past decade in China, this is the first study that attempts to explain the macro factors influencing regular exercise participation. Despite the methodological limitation and concerns about data quality, the main conclusions are in line with existing theories observed in other countries (8, 15) and contribute theoretically to the literature from the world’s second most populous developing country.

## 5 Policy outlook

In developing the National Fitness Plan, our model not only offers a reasonable explanation but also provides the elasticity of the variables. The global model indicates that the economy has a marginal effect of 0.048, whereas university education has a marginal effect of 0.173. Assuming China’s economy continues to grow at an annual rate of 5%, the regular exercise population can increase by 0.24% annually, all else being equal. Based on data from two national population censuses, the proportion of university students relative to the total population increased from 3.71% in 2010 to 7.43% in 2020 (32), representing an annual growth rate of approximately 7.19%. If this growth rate remains constant, the university education will lead to a 1.24% annual increase in the regular exercise population, all else being equal. It is evident that the development of university education is more effective in increasing the regular exercise population. Regarding sports policy, there are two options contingent upon these distinct mechanisms. If the education-health conscientiousness pathway is in play, the National Fitness Plan should prioritize promoting the proactive health concept, particularly by expanding the number of social sports instructors. We propose utilizing government-funded social financing to provide each community with a dedicated social sports instructor (3). However, in the second scenario where the education-employment pathway is in play, there are currently no viable tools for sports policy to influence university education’s impact.

This paper, while focused on sports, underscores the significance of university education in enhancing population quality and boosting China’s global competitiveness. The United States Census Bureau noted in 2021 that 37.9% of Americans aged 25 and older had earned a university degree (44). In contrast, data from the Seventh Population Census shows that only 10.4% of Chinese aged 25 and above hold a university degree (32). As China navigates its economic transition, it appears to have reached the point of diminishing returns with its early WTO-era (2001–2021) economic model, which was fueled by globalization and currency arbitrage. The economic slowdown observed since 2022 suggests that China is still in the process of adapting to new macro-political environments. In an era where efficiency and productivity improvements are increasingly driven by automation, China must shift away from low-quality economic growth to focus on efficiency. This transition is expected to lead to higher and more predictable margins, enhancing China’s economic position globally. Therefore, incorporating the expansion of university education into the 15th Five-Year Plan could provide China with a robust intellectual foundation and support Xi Jinping’s vision of creating new quality productive forces. This move would strengthen the sustainability of its economic development in the post-WTO era, setting the stage for a future where China’s economy is more innovation-driven.

## 6 Conclusion

Using OLS and GWR, we are the first to present evidence that economic development and educational attainment probably drive China’s regular exercise population. We show that the inequalities in the development of the National Fitness Plan also align with the classic Hu Line, with West China generally lagging in regular exercise participation due to these two factors. Considering that higher education influences personal economic prosperity and regional economic development, it can be said that disparities in China’s regular exercise population mirror differences in educational literacy. More importantly, our log–log model for the first time identifies the elasticities of regional GDP and the proportion of the population with a university education, indicating that the marginal effect of university education is more pronounced in the Chinese context. These results hold significant practical value for drafting effective resource allocation policies.

Laozi’s wisdom, “In the pursuit of learning, every day something is acquired” underscores the concept of lifelong education. We contend that access to university education is a fundamental right that should be extended to all young people, beyond the narrow focus on employment outcomes. Such a focus promises to generate a cascading effect, progressively benefiting all facets of Chinese society by fostering a more educated and healthy populace capable of driving innovation, economic resilience, and social well-being.

## Data availability statement

The datasets presented in this study can be found in online repositories. The names of the repository/repositories and accession number(s) can be found at: https://doi.org/10.6084/m9.figshare.25423567.v1.

## Author contributions

Agudamu: Data curation, Formal analysis, Writing – original draft. TB: Funding acquisition, Writing – review & editing. YZ: Conceptualization, Methodology, Project administration, Supervision, Writing – review & editing.

## Footnotes

^1^https://doi.org/10.6084/m9.figshare.25423567.v1
